# Not All Monstrous Cells Indicate Glioblastoma: A Neuropathological Case Report of Pleomorphic Xanthoastrocytoma Misdiagnoses As Giant Cell Glioblastoma

**DOI:** 10.7759/cureus.33735

**Published:** 2023-01-13

**Authors:** George S Stoyanov, Lilyana Petkova, Bogomil Iliev, Radoslav Georgiev, Yavor Enchev

**Affiliations:** 1 General and Clinical Pathology, St. Marina University Hospital, Varna, BGR; 2 General and Clinical Pathology/Forensic Medicine and Deontology, Medical University of Varna, Varna, BGR; 3 Neurosurgery, Medical University of Varna, Varna, BGR; 4 Radiology and Radiotherapy, Medical University of Varna, Varna, BGR

**Keywords:** neurooncology, pleomorphic xanthoastrocytoma, glioblastoma, glial neoplasms, neuropathology

## Abstract

Pleomorphic xanthoastrocytoma (PXA) is a rare central nervous system malignant neoplasm with a relatively favorable prognosis. As PXA histologically presents with large, multinucleated neoplastic cells, its principal differential diagnosis is giant cell glioblastoma (GCGBM). While there is a significant overlap between the two histologically and the neuropathological diagnosis can be challenging, as well as having some overlap neuroradiologically, the patient prognosis differs significantly, with PXA having a more favorable one. Herein we present a case report of a male patient in his thirties diagnosed with GCGBM and presenting again six years later with thickening of the wall of the porencephalic cyst suggestive of disease recurrence. Histopathology revealed neoplastic spindle, small lymphocyte-like, large epithelioid-like, some with foamy cytoplasm, and scattered large multinucleated cells with bizarre nuclei. For the most part, the tumor had a distinct border to the surrounding brain parenchyma, except for a single zone of invasion. As per the depicted morphology, with a lack of pathognomic features of GCGBM, the diagnosis of PXA was defined, and the oncologic committee reevaluated the patient with treatment reinitiation. Based on the close morphological profile of these neoplasias, it is likely that in the case of limited material, multiple PXA cases are diagnosed as GCGBM, resulting in misdiagnosed long survivors.

## Introduction

Neuropathology, as a subfield of pathology, relies on a specific set of modalities and a combination of clinical, neuroradiological, pathomorphological, immunophenotypical, and molecular data for diagnosis [1]. Like in all fields of pathology, however, the key in these is the microscopic histopathological evaluation of samples. In the field of neoplastic neuropathology, sufficient such material is vital, as pathognomonic findings may not be present in minimal material sent for histopathology [1].

Such classical pathognomic signs are glomeruloid microvascular proliferation and palisading necrosis for glioblastoma (GBM), prior to the implementation of the 2021 and especially the 2016 World Health Organization (WHO) classification of nervous system tumors [2]. GBM, often referred to as the great imitator in neuropathology, classically has multiple histological variants, such as conventional, epitheloid, gliosarcoma, granular cell, small cell, and giant cell GBM [2].

Giant cell GBM (GCGBM) is a distinct morphological entity without solid genetic data pointing towards unique molecular mechanisms of development [2]. Typical for this entity is the dominant cellular component of giant multinucleated tumor cells, classically referred to as monstrous cells, often among a reticulin-rich stroma, often with as much as 20 or more nuclei [1,2]. While most GBM samples have a varying amount of giant cells, GCGBM requires these to be the dominant cellular fraction. The diagnosis, however, requires careful interpretation of the whole specimen for other GBM pathognomonic signs, as other rare gliomas can mimic these cells [1].

One such entry is pleomorphic xanthoastrocytoma (PXA) - a lower WHO-grade astrocytic neoplasm with varying admixtures of giant cells and reticulin stroma, which falls into the group of circumscript (well-demarcated, non-invasive gliomas) and does not harbor microvascular proliferation or spontaneous necrosis [1-5]. Based on the significant overlap between the two, it is likely that a significant number of PXA are diagnosed as GCGBM and further reported as long survivors.

## Case presentation

A previously healthy male patient in his thirties presented to our institution with complaints of progressive headache, nausea, vomiting, gait instability, and somnolence developing over the previous two weeks. Outpatient neuroradiology established a relatively well-demarcated tumor formation in the right parietooccipital area with perifocal edema measuring up to 50 mm in its greatest diameter. The findings suggested astrocytoma, primary central nervous system (CNS) lymphoma, or GBM.

The patient was scheduled for emergency neurosurgical intervention for decompression as in-hospital neuroradiology confirmed the findings, but also noted compression, mediation, and dilation of the ventricular system. During surgery, the tumor was found to be grayish, relatively well-demarcated, and richly vascularized but did not bleed spontaneously. A temporary ventricular shunt was placed for ventricular decompression, and the tumor was excised to an optimal extent - gross total resection. Specimens were sent for frozen section neuropathology, suggesting a high-grade glial neoplasm rich in multinucleated giant cells. The postoperative period was uneventful.

The specimen sent for histopathology was fragmented, rich in giant cells amidst a reticulin-rich stroma. Tumor cells stained positive for glial fibrillary acidic protein (GFAP) and had a Ki-67 index of more than 30%. Hence the diagnosis of GBM, WHO grade 4, was established.

The patient was presented to the oncologic committee and received six cycles of temozolomide, which were well tolerated, and volumetric modulated arc therapy to a total of 60 Grays (Gy), split up into 2 Gy doses. The neurological status of the patient slowly improved, and he was regular to follow-up, with no newly developing complaints; neuroradiology showed minimal suspicions of residual tumor in the periphery of the post-tumorectomy cyst.

**Figure 1:**
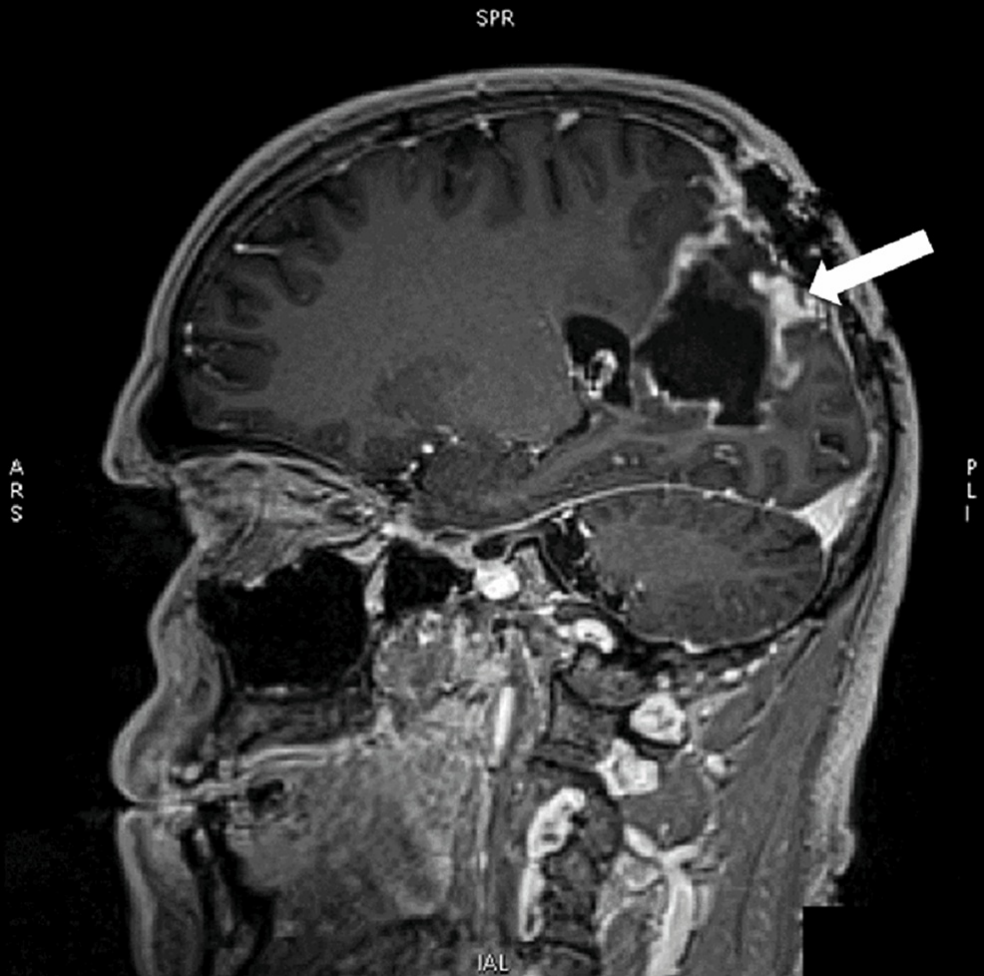
SAG 3D T1+C MRI of the brain showing postoperative changes with right parietal porencephalic cyst and peripheral zones of contrast enhancement, suggestive of residual tumor (arrow) SAG 3D T1+C MRI: sagittal three-dimensional longitudinal relaxation time contrast-enhanced magnetic resonance imaging

Six years after the surgery, the patient was again referred to our institution by the monitoring outpatient neurologist due to noted neuroradiological thickening of the cyst wall and intermittent headaches (Figure 1). Based on the clinical and neuroradiological findings - suspicion of disease recurrence, the patient was scheduled for a reoperation. During surgery, the cyst's wall was noted to be thick and had the same characteristics as the tumor previously excised. A gross total resection was performed again. The postoperative period was uneventful.

**Figure 2:**
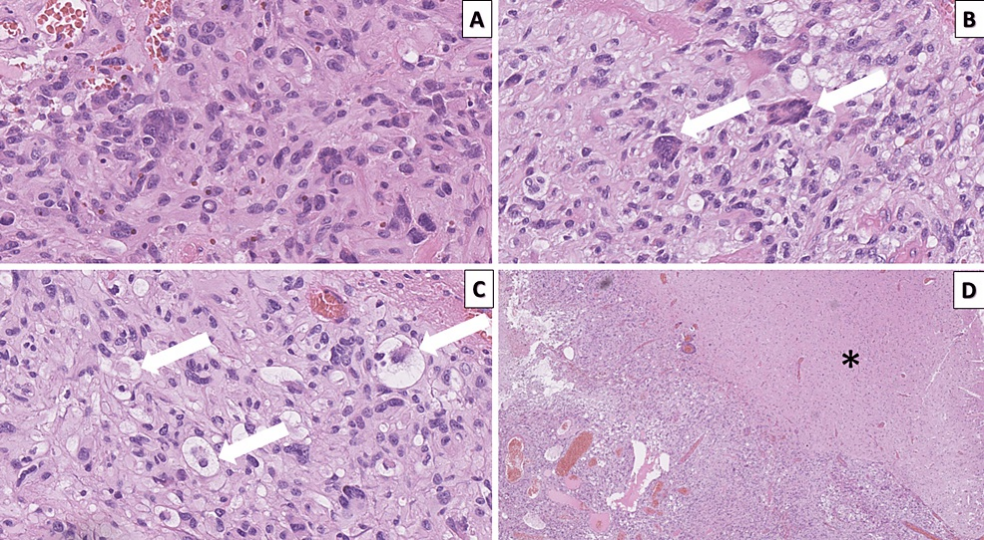
Histopathology of the tumor A: cellular pleomorphism, H&E stain, original magnification x400; B: bizarre and monstrous cells (arrows), H&E stain, original magnification x400; C: xanthomatous cells (arrows), H&E stain, original magnification x400; D: the sharp border between the tumor and the surrounding non-infiltrated brain parenchyma (asterisk), H&E stain, original magnification x4. H&E: hematoxylin and eosin

The specimen sent for histopathology was fragmented, soft, and yellowish with focal hemorrhagic areas. Histopathology revealed brain parenchyma involved by a tumor proliferation represented by spindle and polymorphic atypical cells, small lymphocyte-like, large epithelioid-like, some with foamy cytoplasm, others with eosinophilic granular cytoplasmic inclusions and scattered large multinucleated cells with bizarre nuclei (Figure 2). For the most part, the tumor formation has a sharp and distinct border compared to the surrounding parenchyma, without phenomena of satellitosis, single zones of growth to the surrounding parenchyma from the spindle, and lymphocyte-like tumor cells. Extensive areas of tumor necrosis and high mitotic activity, significantly over the required > 5 per 10 high-power fields, with the presence of atypical mitotic figures. Abundant lymphoid infiltration in the tumor mainly clustered perivascularly. An abundance of thick-walled vessels with degenerative changes in the wall, siderophages, and a peripheral group of foamy macrophages among reactive gliosis, most likely an expression of the previous neurosurgical intervention in the tumor area.

Based on the histological findings, rather than GCGBM, a diagnosis of PXA, WHO grade 3 was established, and further molecular analysis was suggested for v-RAF murine sarcoma viral oncogene homolog B1 V600E analog (BRAF V600E) and Cyclin-dependent kinase inhibitor 2A/B (CDKN2A/B).

Almost half a year after surgery, the patient is recovering well, with no new onset symptoms, and is regularly to follow up.

## Discussion

PXA is a relatively rare CNS tumor, with a yearly incidence of around 0.7 cases per 100,000 capita, representing less than 0.3% of all primary CNS tumors [3,6]. The overlap of histological criteria between PXA and GCGBM, mainly the giant multinucleated cells, reticulin-rich stroma, and admixture of other glial cellular components, make the differential diagnosis between the two challengings [1-3]. Classically, however, GCGBM has pathognomonic glomeruloid microvascular vascular and palisading/pseudopalisading necrosis. PXA, on the other hand, lacks these features, and while both tumors are classically relatively well-demarcated neuroradiologically from the surrounding parenchyma, only PXA does not show areas of direct invasion and histologically has an admixture of foamy cells, rarely seen in GBM [3,7].

In our case, based on the brisk mitotic activity, the WHO CNS grade of 3 was placed, which was likely the case of grade in the initial diagnosis as well, as the Ki-67 index of more than 15%, as per the current classification, always warrants this grade [3]. While most PXA present initially as WHO CNS grade 2, a transition to WHO CNS grade 3 is typical in case of recurrence [3].

Furthermore, as underlined by our case, PXA has a significantly more favorable prognosis than GBM [8,9]. While the five-year survival rate for WHO CNS grade 2 PXA is 90.4% and for WHO CNS grade 3 PXA it is 57.1%, the rate for GBM is only 7-10%, with GCGBM having an overall better prognosis than conventional GBM with a median survival of little more than a year [2,3].

Histopathological differentiation is not aided by immunohistochemistry, as both tumors are within the same histogenic group of astrocytic gliomas and are hence positive for GFAP; the Ki-67 index, which aids in the grading of multiple histologically different malignant neoplasia, would also be of little aid as GBM can have an exceedingly small Ki-67 proliferative index [10]. Testing for isocitrate dehydrogenase (IDH) mutational status, both on immunohistochemistry and via molecular analysis, would prove helpful, as GBM is always IDH-wild type as per the 2021 WHO classification of nervous system tumors, although there may overlap with some mutations, especially in WHO grade 4 IDH-mutant astrocytomas [11]. The highest significance in differential expression is for the p53 protein, where mutant variants are rare in PXA but common in GBM [2,3,11]. Therefore the differential between PXA and GCGBM is, to a large extent, a morphological one, as neuroradiology also has significant overlap between the two entries and is definitive in larger specimens, where no vascular proliferation and necrosis are present, on the background of a sharp demarcation with the surrounding parenchyma [2,3].

Based on the significant morphological overlap between PXA and GCGBM it is likely that the true incidence of PXA is higher, as in cases akin to ours where the histopathology specimens are fragmented and limited, cases are reported as GCGBM. This feature would be of interest to the patient - more intensive treatment allowing for better survival and intensive monitoring, as in our case. These cases would however also increase the reported survival in GBM and further be reported as long survivors while belonging to another neoplastic subgroup.

## Conclusions

PXA is a rare neoplasia of the CNS, a member of the circumscribed glioma group. Although there is a significant overlap between the histological diagnostic criteria with GCGBM and the neuroradiological parameters, there is a substantial difference in patient prognosis, with PXA having a significantly more favorable one.

As underlined by our case, in limited specimens for histopathological evaluation, the diagnosis can be challenging and, based on the epidemiological discrepancies between the two, suggestive of GCGBM. Significant postoperative survival, especially in the context of neuroradiological evidence of residual tumor, should warrant revision of the diagnosis, as these cases are further included as long GBM survivors and change the survival characteristics of the disease.
